# Deaf education in Croatia

**DOI:** 10.3325/cmj.2013.54.89

**Published:** 2013-02

**Authors:** Iva Rinčić, Amir Muzur

**Affiliations:** Department of Social Sciences and Medical Humanities, School of Medicine, University of Rijeka, Rijeka, Croatia

In 2011, in Croatia there were 13 230 persons with impaired hearing and 2763 out of them had total or major disability (1). Their communication through the classical means of writing and reading is sometimes greatly hampered by their modest vocabulary and agrammatism, and they are often victims of prejudice.

Croatia has a rich tradition of activities aimed at integration of the deaf. The Croatian Association of the Deaf and Hard of Hearing was established in 1921 and today has approximately 9000 members in 23 organizations. This association is a member of the World Federation of the Deaf (WFD) and the International and European Federation of Hard of Hearing People, and it is also affiliated to the national Association of Organizations of Disabled Persons in Croatia (2). As early as 1955, the World Federation of the Deaf organized its second congress in Zagreb, which was attended by approximately 2000 deaf people and experts in the field from around the world. On that occasion, the idea to organize the International Day of Deaf Persons was initiated, and the President of the Yugoslav Association, Dragoljub Vukotić (1924-1997) became president of the Federation.

In modern Croatia, the key document that provided the strategic framework for the education of the deaf at the national level was the National Strategy of Equal Opportunities for Persons with disabilities 2007-2015, which was published by the Government of the Republic of Croatia in 2007, especially Fields of Activities 2.2 Life in the Community and 2.3 Upbringing and Education (3). The international framework was mapped out primarily by the Recommendation 1598 of the Council of Europe on the protection of sign languages of 2003, especially Article 10, which encouraged member states to include sign languages as a valid academic qualification and to subsidize the publication of instructive literature in sign languages (4), and of course, by the UN Convention on the Rights of Persons with Disabilities of 2006, especially the Article 24 on Education (5). Unfortunately, to implement these recommendations, it is necessary to educate interpreters. At the moment, there are about twenty of them in Croatia, three of whom are in Zagreb. The Croatian Sign Language Act, although written, has still not been sent to the adoption procedure (6).

One of the most important institutions for helping people with hearing loss and impairment is The Department for Hearing Impairment of the University of Zagreb School of Education and Rehabilitation Sciences. The Department emerged from the High School for Special Needs (established in 1962) and its activities include studying the grammar of the Croatian sign language and postgraduate specialist studies in deaf education.

The SUVAG Polyclinic (*Systeme universel verbotonal d'audition Guberina*) was established in 1961 and it has been dedicated to studying the pathology of hearing and/or speech and to the verbotonal rehabilitation method. The founder of the Polyclinic was Petar Guberina (1913-2005), Professor at the University of Zagreb, Head of the Department for Romance Studies, founder of the Institute (later Department) for Phonetics, and member of the Croatian Academy of Sciences and Arts. In the mid-1950s, Guberina established a theory according to which it is possible to rehabilitate speech or hearing by stimulating the remaining auditory capacities. After precise determination of the location of the impairment, the residual capacity rehabilitation is achieved through the stimulation of all spacioceptive systems: stereognosis, stereophonics, sensomotorics, etc. The method developed special equipment (SUVAG, VERBOTON) and diagnostics (7), and it was accepted in many centers worldwide (eg, The Hearing & Speech Foundation, Maryville, TN, USA) (8). According to its own statistics, 85% of children from the SUVAG Polyclinic continued education in regular schools, and 11% went to high school or university (9).

In 2002/2003, at the Rijeka School of Medicine, Ivan Šegota established an elective course entitled How to Communicate with Deaf Patients. The idea to start educating future physicians and nurses on communication with deaf patients was inspired by a case mentioned by Šegota in his book: “…there was a deaf patient in Rijeka whom, even after several days in hospital, none of the nurses or physicians even tried to approach. His son, who was also my student, told me about this in the bioethics exam, and said that his father was frightened and anxious because he was being examined and subjected to blood tests and various therapies without knowing what illness he had, what his chances of recovery were, and how long he had to stay in the hospital” (10). Since he was concerned with the bioethical doctrine of informed consent as the central issue in the relationship with deaf patients, Šegota, after contacting Damir Herega, TV interpreter for sign language, started in 2000/2001 an “informal and optional course of sign language for all interested students of the Rijeka School of Medicine, organized by the CROMSIC student association” (10). In the following period, he elaborated the idea about communication in sign language as the “only correct solution” to achieve privacy in communication with patients in a (bio)ethical sense (10). His vision about the systematic education of students on communication with deaf patients was crucial for future educational and scientific efforts in this field (11,12).

In 2007 Ivan Šegota started the project Clinical Bioethics: Education for Communication with Deaf Patients at the University of Rijeka School of Medicine. The project introduced the basics of communication with deaf patients into the “Rijeka Model of Bioethical Education,” with the final aim to move closer to European trends by introducing sign language into Croatian health care, by educating physicians and nurses.

The book The Deaf and Medical Sign Language: How to Communicate with Deaf Patients (13) by Ivan Šegota and associates was published in 2010. By standardizing and introducing new medical terms in sign language, this book enriched Croatian sign language, making it one of the most advanced in Europe. Subsequently, a significant improvement in the quality of life for the deaf and hard of hearing in Rijeka and the region has been noticed.

Further development of scientific structure in the field dealing with deaf people is expected. It is important to cover all aspects of communication in the doctor-patient relationship. However, even in such communication deaf patients are put in an unequal position: their participation in communication depends on the physician's readiness to communicate in sign language. In other words, the problem of the relationship between the health worker and deaf patient involves much more than just communication in sign language.

Rijeka School of Medicine since recently provides not only education for communication with deaf patients, but strategic institutional education for the deaf to enable them to enter the academic world, open up new employment opportunities, provide more information, and to help overcome numerous barriers in their daily living.
